# Correlation between miR-148 Expression in Vitreous and Severity of Rhegmatogenous Retinal Detachment

**DOI:** 10.1155/2017/3427319

**Published:** 2017-02-05

**Authors:** Taichi Tsunekawa, Hiroki Kaneko, Kei Takayama, Shiang-Jyi Hwang, Akio Oishi, Yosuke Nagasaka, Fuxiang Ye, Takeshi Iwase, Norie Nonobe, Shinji Ueno, Yasuki Ito, Shunsuke Yasuda, Toshiyuki Matsuura, Hideyuki Shimizu, Ayana Suzumura, Keiko Kataoka, Hiroko Terasaki

**Affiliations:** ^1^Department of Ophthalmology, Nagoya University Graduate School of Medicine, Nagoya, Japan; ^2^Laboratory of Bell Research Center-Department of Obstetrics and Gynecology Collaborative Research, Nagoya University Graduate School of Medicine, Nagoya, Japan; ^3^Department of Ophthalmology and Visual Sciences, Kyoto University Graduate School of Medicine, Kyoto, Japan; ^4^Department of Ophthalmology, Shanghai First People's Hospital, Shanghai Jiao Tong University School of Medicine, Shanghai, Japan

## Abstract

*Purpose.* We had earlier reported positive hsa-miR-148a-3p expression in eyes with rhegmatogenous retinal detachment (RRD) and its involvement in the epithelial-mesenchymal transition of retinal pigment epithelium in vitro. Here we investigated the association of hsa-miR-148a-3p expression levels in the vitreous fluid of patients with RRD with severity of RRD.* Methods.* The hsa-miR-148a-3p expression levels in the vitreous fluid, range (degree) of retinal detachment (RD), and pixels of retinal break were measured in 27 eyes with RRD. The association of hsa-miR-148a-3p expression levels with other factors was evaluated by multiple regression analysis.* Results.* The hsa-miR-148a-3p expression levels, time from onset of RRD to vitrectomy, range of RD, and pixels of retinal breaks were 23.68 ± 43.00, 12.07 ± 15.36 days, 155.85 ± 86.67 degrees, and 37000 ± 67100 pixels, respectively. Five eyes with RRD had vitreous hemorrhage preoperatively. The hsa-miR-148a-3p expression levels were significantly associated with pixels of retinal breaks (*β* = 0.699) and the time from onset of RRD to vitrectomy (*β* = 0.358) but not with the range of RD or presence of vitreous hemorrhage.* Conclusion.* The hsa-miR-148a-3p expression levels in the vitreous fluid were significantly associated with the size of retinal break and disease duration.

## 1. Introduction

Retinal detachment (RD) is a serious disease that can cause blindness [1]. The incidence of RD was reported to be 0.6–1.8 per 10000 people [2], and the most common type of RD is rhegmatogenous RD (RRD), which is mainly caused by retinal breaks owing to vitreous traction [3]. Despite recent advances in surgical methods and technology that have improved the rates of structural recovery following surgery, [4, 5] it is difficult to recover all cases of RRD completely. Patients with severe and/or long-standing RRD or those with unsuccessful surgical treatment tend to develop proliferative vitreoretinopathy (PVR), which renders the surgical management more challenging [6, 7].

Several studies have sought to elucidate the clinical and biological causes of PVR. The extent of detachment, long-standing RD, presence of vitreous hemorrhage, large retinal breaks, and presence of inflammation were reported as risk factors for the development of PVR [8–12]. Several biological factors were previously reported to be implicated in the pathogenesis of PVR, and retinal pigment epithelium (RPE) cells were considered the key element [8]. In RRD, the changes that occur after RPE cells lose contact with photoreceptors can be the trigger mechanism for the onset of PVR. RPE cells are present in proliferative membranes and exhibit different morphologic characteristics, such as macrophage-like and fibroblast-like, in the development of PVR [13]. In addition, TGF-*β*1 can modify the phenotype of RPE cells with changes in the expression of *α*-smooth muscle actin and fibronectin synthesis [14]. In RRD, RPE cells float into the vitreous cavity through the retinal breaks, adhere to the surface of the sensory retina, and undergo transformation from epithelial to mesenchymal cells. Transformed RPE cells migrate, proliferate, and transform into fibroblasts, leading to PVR [15]. This biological phenomenon is referred to as the epithelial-mesenchymal transition (EMT) [16–19].

MicroRNAs are small noncoding single chain RNA transcripts which comprise 21–24 nucleotides that regulate various cellular processes by modulating mRNA and protein levels [20, 21]. In the human body, more than 2000 microRNAs are reportedly involved in cell proliferation, differentiation, cell fate determination, signaling, organ development, and cellular responses to viral infection. These regulate approximately 90% of all cellular processes, including tumor formation, and have been linked to a number of human diseases [22–25]. The role of mRNAs as therapeutic targets or as disease markers has been an active area of research [22–25]. In the eye, various microRNAs are thought to act on the retina or on the RPE and play important roles in neuroprotection and angiogenesis [17, 26–29].

In a recent study, we detected hsa-miR-148a-3p in vitreous and subretinal fluid in eyes with RRD but not in those with macular hole. Furthermore, hsa-miR-148a-3p was shown to promote EMT of RPE in vitro [30]. These results indicate that hsa-miR-148a-3p promotes PVR in RRD eyes via enhancing EMT of RPE cells. However, whether enhanced expression of hsa-miR-148a-3p in eyes with RRD is clinically related with the pathogenesis of PVR is not known.

In this study, we investigated the potential correlation of increase in hsa-miR-148a-3p expression in RRD eye with known clinical risk factors for PVR.

## 2. Materials and Methods

### 2.1. Patients and Sample Collection

Twenty-seven vitreous samples were collected from patients with RRD and nine vitreous samples were collected from patients with PVR. All samples were collected by dry vitrectomy using a vitrectomy cutter before starting infusion and were immediately stored at −80°C. The presence of vitreous hemorrhage was evaluated prior to surgery.

Patients with severe systemic diseases, for example, autoimmune diseases and cancer, or those with a history of vitrectomy were excluded. The present study adhered to the guidelines of the Declaration of Helsinki; the study was approved by the Nagoya University Hospital Ethics Review Board. Written informed consent was obtained from all patients prior to their enrolment.

### 2.2. Classification of PVR

PVR was diagnosed and classified according to the previous criteria [31]; Grade A is described by vitreous haze, vitreous pigment clumps, and pigment clusters on the inferior retina; Grade B is described by the wrinkling of the inner retinal surface, retinal stiffness, vessel tortuosity, rolled and irregular edge of retinal breaks, and decreased mobility of vitreous; Grade CP is described by focal, diffuse, or circumferential full-thickness folds and subretinal strands posterior to equator; Grade CA is described by focal, diffuse, or circumferential full-thickness folds and subretinal strands anterior to equator, anterior displacement, and condensed vitreous with strands. Type 1 is focal; type 2 is diffuse; type 3 is subretinal; type 4 is circumferential; and type 5 has anterior displacement.

### 2.3. Isolation and Real-Time Quantitative PCR for microRNA

The protocols for RNA isolation and qPCR are described elsewhere [30]. Vitreous fluid was thawed slowly, centrifuged at 3,000 ×g for 5 min at 4°C to exclude cell debris, and stored prior to use in further experiments. Total RNA was extracted from supernatants using Qiagen miRNeasy® Mini Kits (Qiagen, GmbH, Hilden, Germany), according to the manufacturer's recommendations. For normalization of sample-to-sample variation during RNA isolation procedures, 25 fmol of synthetic* Caenorhabditis elegans* miRNA cel-miR-39 was added to total RNA samples, dissolved in RNase-free water, and stored at −80°C. qPCR was performed to confirm the upregulation of candidate microRNA transcripts detected on microRNA microarray.

For measurement of microRNA expression levels, specific primer against hsa-miR-148a-3p was used and its expression quantified on TaqMan miR assays (Applied Biosystems, Foster City, CA), according to the manufacturer's recommendation, using an MX3000p instrument (Stratagene, La Jolla, CA). The number of miRNA copies was normalized using stably expressed RNU44 small nucleolar RNA.* Caenorhabditis elegans* miR-39 (cel-miR-39) was added and its expression measured to confirm the stability of the experimental processes. The expression levels of hsa-miR-148a-3p were determined using the 2^−ΔΔCt^ method as relative expression [32].

### 2.4. Measurement of Range of RD and Size of Retinal Break

**Figure 1:**
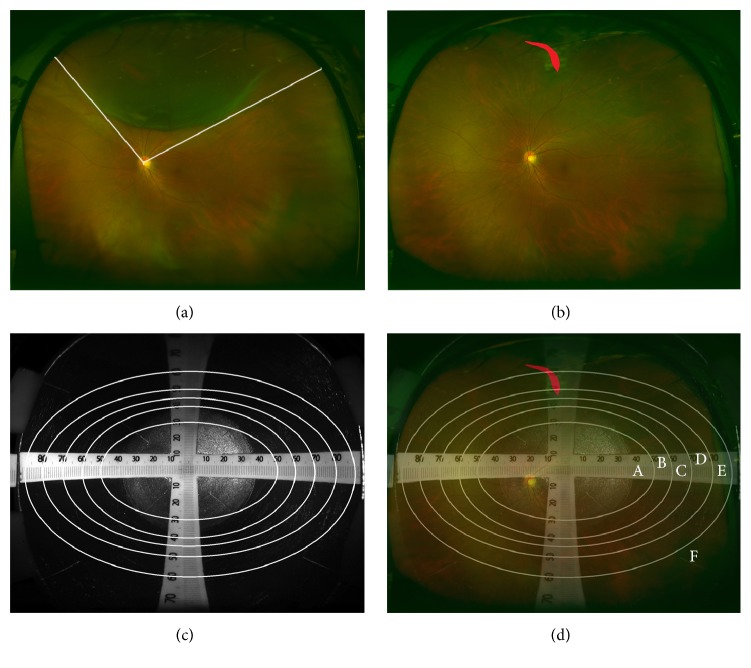
Measurement of the pixels in retinal break and the range of RD. (a) Representative preoperative fundus image from 55-year-old male patient with RRD obtained with ultra-wide-field scanning ophthalmoscope. The angle of RD was measured by the two lines starting from the optic disc. (b) The postoperative fundus image from the same patient captured by ultra-wide-field scanning ophthalmoscope. The retinal break is filled in red. (c) The fundus image of the simulated eye with scale obtained with ultra-wide-field scanning ophthalmoscope. The circles delineating the areas with the same size were designed based on the scale. (d) Merged image of (b) and (c). Total pixels of the retinal break (filled in red) were corrected based on the ratio of each area (A–F). RRD, rhegmatogenous retinal detachment; RD, retinal detachment.

Retinal breaks and RD were imaged by ultra-wide-field scanning ophthalmoscope (Optos, Marlborough, MA, USA) before and after surgery. Figures 1(a) and 1(b) are representative fundus images before and after surgery from the same patient with RRD. The range of RD was measured as the angle of RD range around the optic disc in the fundus image obtained on preoperative ultra-wide-field scanning ophthalmoscope (Figure 1(a)). The pixels of the retinal breaks were measured with ImageJ [33]. In case of multiple retinal breaks, the total value of the retinal breaks was applied. Super-wide-angle fundus photograph is useful for measuring pixels in the retinal break, but the most peripheral part of the image has been reported to be significantly and unequally magnified [34]. Therefore, to adjust for the difference in the actual size and fundus image, we first prepared the fundus image from the simulated eye with the scale captured by the ultra-wide-field scanning ophthalmoscope (Figure 1(c)) and calculated the gap of the actual scale and the scale in the photoimage. The fundus image was divided into six areas depending on the difference of the magnification (Figure 1(d)). The corrected pixels in the retinal break were calculated as total pixels corrected for each area (A–F in Figure 1(d)).

The equation used was as follows:

pixels in A + $\frac{pixels\;in\;B}{1.2}$ + $\frac{pixels\;in\;C}{1.3}$ + $\frac{pixels\;in\;D}{1.45}$ + $\frac{pixels\;in\;E}{1.\;6}$ + $\frac{pixels\;in\;F}{2.0}$.

### 2.5. Statistics

Data are expressed as mean ± standard deviation. Multiple regression analysis was used to evaluate the correlation between the expression levels of hsa-miR-148a-3p and the independent variables such as the corrected pixels of the retinal break, the range of RD, and the time from onset from RRD to vitrectomy.

With regard to the presence of vitreous hemorrhage, data was statistically analyzed using the Mann–Whitney *U* test (unpaired samples); associated *P* values < 0.05 were considered indicative of a statistically significant between-group difference. Spearman's rank correlation coefficient was used to assess the association between expression levels of hsa-miR-148a-3p of PVR and the time from onset of RRD to vitrectomy.

## 3. Results

### 3.1. Characteristics of Patients with RRD

Twenty-seven patients (27 eyes) with RRD were included in the study. Mean age of patients was 58.40 ± 12.93 years; the mean time from onset of RRD to vitrectomy was 12.07 ± 15.36 days. Corrected pixels in retinal break were 37000 ± 67100 pixels, the range of retinal detachment was 155.85 ± 86.67 degrees, and the expression levels of hsa-miR-148a-3p were 23.67 ± 43.00 (Table 1). Five eyes with RRD had preoperative vitreous hemorrhage.

### 3.2. Relationship between hsa-miR-148a-3p and the Clinical Parameters of RD

**Figure 2:**
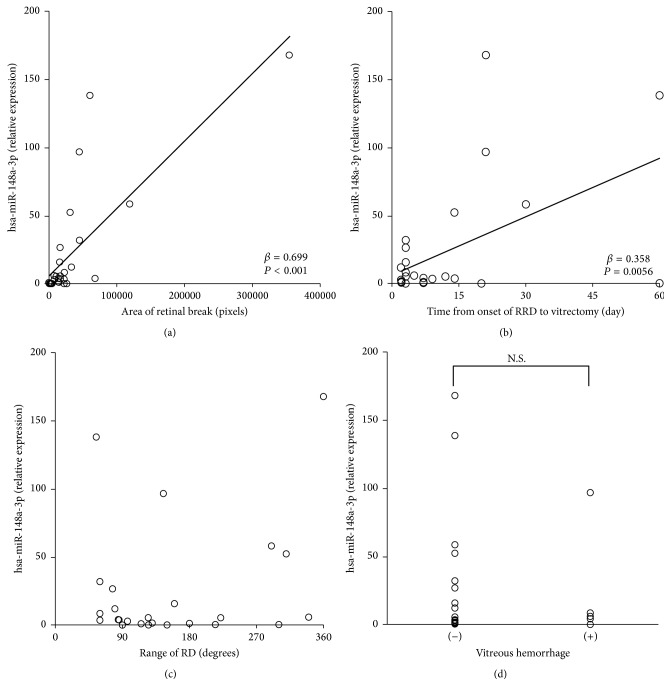
Relationship between hsa-miR-148a-3p expression and clinical parameters of RD. Open circles represent the values in each case (a, b, c, and d). The expression level of hsa-miR-148a-3p showed significant correlations with the area of the retinal break and the time from onset of RRD to vitrectomy. (c) The expression levels of hsa-miR-148a-3p did not show significant correlations with the range of the RD (*P* = 0.985). (d) The existence of the vitreous hemorrhage did not show significant difference in hsa-miR-148a-3p expression level (*P* = 0.661). RRD, rhegmatogenous retinal detachment; RD, retinal detachment; N.S., not significant.

On multiple stepwise linear regression analyses (Table 2), a significant association of the expression levels of hsa-miR-148a-3p with corrected pixels of retinal break (*β* = 0.699; *P* < 0.001) and the time from onset of RRD to vitrectomy (*β* = 0.358; *P* = 0.0056) was observed. However, no significant association of hsa-miR-148a-3p expression with the range of RD and presence of vitreous hemorrhage was observed. The scatter plot of the association between hsa-miR-148a-3p expression and other parameters is presented in Figure 2. Corrected pixels of retinal break showed the strongest correlation with the expression level of hsa-miR-148a-3p (Figure 2(a)). The presence of the vitreous hemorrhage did not show a significant association with the hsa-miR-148a-3p expression level (*P* = 0.661; Figure 2(d)).

### 3.3. Patients with Proliferative Vitreoretinopathy and the Expression Levels of hsa-miR-148a-3p

**Figure 3:**
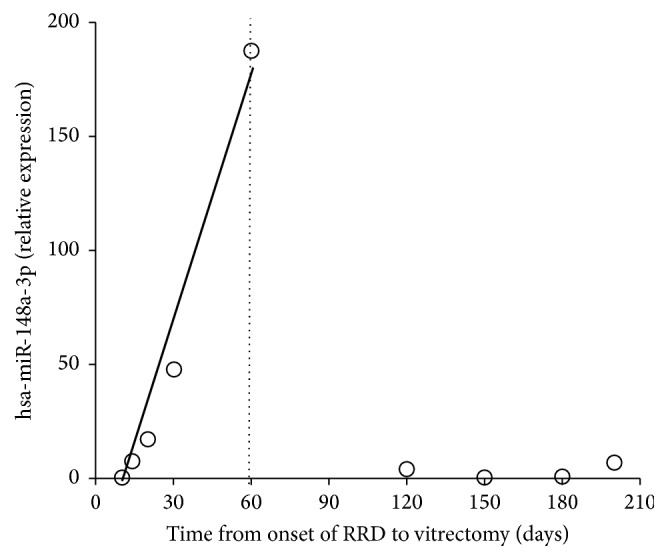
Expression levels of hsa-miR-148a-3p in proliferative vitreoretinopathy (PVR) and time from onset of RRD to vitrectomy. In patients with PVR that underwent vitrectomy within 2 months after the onset of RRD (based on patient declaration), the expression levels of hsa-miR-148a-3p showed a significant correlation with time from onset of RRD to vitrectomy (*r* = 1.0, *P* < 0.01). Low expression levels of hsa-miR-148a-3p were observed in patients who underwent surgery ≥ 60 days after the onset of RRD. RRD, rhegmatogenous retinal detachment.

A total of nine patients (9 eyes) had PVR. PVR severity was assessed based on the classification system reported elsewhere (Table 3) [31]. Among patients who underwent surgical vitrectomy within 2 months after the onset of RRD (based on patient declaration), the expression levels of hsa-miR-148a-3p showed a significant correlation with the time from onset of RRD to vitrectomy (*r* = 1.00; *P* < 0.01; Figure 3). On the other hand, expression levels of hsa-miR-148a-3p in patients who underwent surgery ≥ 60 days after the onset of RRD were extremely low (Figure 3).

## 4. Discussion

Hsa-miR-148a has been shown to have multiple functional roles depending on the cellular conditions. In hepatocellular carcinoma cells, for example, increase in hsa-miR-148a-3p promoted cell proliferation, cell cycle progression, cell migration, anchorage independent growth in soft agar, and subcutaneous tumor formation [35]. In aortic valve interstitial cells, hsa-miR-148a decreased nuclear factor kappa-light-chain-enhancer of activated B cell (NF-*κ*B) signaling and NF-*κ*B target gene expression [36].

In a translational research in ophthalmology, we detected hsa-miR-148a-3p in vitreous fluid and SRF in eyes with RRD. Administration of hsa-miR-148a-3p was found to promote EMT of RPE [30], which was confirmed by the upregulation of *α*-smooth muscle actin expression and increased migration ability of two different types of human RPE cells. Based on the scientific background that EMT of RPE is relevant to the pathogenesis of PVR, these findings suggest an association between the upregulation of hsa-miR-148a-3p and PVR.

The extent of detachment, large size of retinal break, prolonged RD, vitreous hemorrhage, and intraocular inflammation were shown to be risk factors for PVR in RRD eyes [8–12]. In cases of RD with a large retinal break, a greater number of RPE cells are dispersed into the vitreous. In patients with long-standing RD, RPE cells are exposed to the vitreous body over a prolonged period which allows the spread of RPE cells into the vitreous space. The reported average period between the onset of RRD and the development of PVR is 2.0 months (range: 0.5–45 months) [37]. Scientifically, it is difficult to assess that PVR develops immediately after the onset of RRD. This is because the underlying biological change requires certain period until the involved cells show migration, transformation, and increased proliferation. Vitreous hemorrhage introduces serum components into the vitreous cavity and this probably enhances RPE cell proliferation, which results in PVR development [38–40]. However, we previously demonstrated that hsa-miR-148a-3p levels in the blood serum were lower than intraocular hsa-miR-148a-3p levels in patients with RRD [30]. Therefore, it appears that hsa-miR-148a-3p in the vitreous hemorrhage that is originally from the serum may be less important in the pathogenesis of PVR.

In the present study, increased expression levels of hsa-miR-148a-3p showed significant correlations with the time from onset of RRD to vitrectomy and presence of large retinal breaks. However, one of the limitations of our study is that the sample size, especially of PVR, was quite small and the standard deviation of the retinal break was large. It is better that the clinical and scientific correlation between hsa-miR-148a-3p and PVR is obtained from larger number of samples. Nevertheless, we do not often have PVR cases and, thus, it is difficult for us to do the power calculation. Further studies with larger number of samples should be conducted. The other limitation is that there was no control group in this study, although hsa-miR-148a-3p expression was not detected in the vitreous samples obtained from eyes with the other retinal diseases, for example, macular hole and vitreomacular traction syndrome [30]. Therefore, it was theoretically very difficult to settle absolute control for this analysis. In addition, we were unable to elucidate the detailed mechanisms that underlie the promotion of PVR via EMT by hsa-miR-148a-3p. Further studies are required to investigate the source of the secreted hsa-miR-148a-3p into the vitreous.

In conclusion, increased expression levels of hsa-miR-148a-3p which promotes EMT in RPE cells correlated with the time from onset of RRD to vitrectomy and the size of retinal breaks in eyes with RRD. Accumulation of knowledge related to microRNAs will expand therapeutic possibilities for intractable diseases such as PVR. Further research is required to elucidate the biological and clinical importance of microRNA in many human diseases.

## Supplementary Material

The results of multiple regression analyses excluding the cases with vitreous hemorrhage are shown in supplementary Table.

## Figures and Tables

**Table 1:** Characteristics of patients with RRD.

Parameters	Mean ± SD
Age (years)	58.41 ± 12.93
Time from onset of RRD to vitrectomy (days)	12.07 ± 15.36
Range of RD (degrees)	155.85 ± 86.67
Area of retinal break (pixels)	37000 ± 67100
hsa-miR-148a-3p (relative expression)	23.68 ± 43.00

SD, standard deviation; RRD, rhegmatogenous retinal detachment; RD, retinal detachment.

**Table 2:** Correlation between the expression levels of hsa-miR-148a-3p and clinical parameters in patients with RRD.

Parameter	*β*	*P* value
Area of retinal break (pixels)	0.699	<0.001
Time from onset of RRD to vitrectomy (days)	0.358	0.0056
Vitreous hemorrhage	0.110	0.344
Range of RD (degrees)	0.0024	0.985

RRD, rhegmatogenous retinal detachment; RD, retinal detachment.

**Table 3:** Characteristics of patients with PVR.

Case	Age (years)	Time from onset of RRD to vitrectomy (days)	Vitreous hemorrhage	Grade	Type	hsa-miR-148a-3p (relative expression)
#1	40	10	−	B		0.37
#2	30	14	−	CP	3	7.48
#3	53	20	−	CP	3	16.97
#4	14	30	−	CP	3	47.67
#5	43	60	−	CA	3	187.4
#6	17	120	−	CP	3	4.05
#7	15	150	−	CA	4	0.46
#8	44	180	−	CA	4	0.68
#9	68	200	+	CP	1	6.64

PVR, proliferative vitreoretinopathy; RRD, rhegmatogenous retinal detachment.
