# Supplementary material for: Correlation between miR-148 Expression in Vitreous and Severity of Rhegmatogenous Retinal Detachment
Source: Biomed Res Int. 2017 Feb 5;2017:3427319. doi: 10.1155/2017/3427319 (PMC5316437; doi:10.1155/2017/3427319)
Supplement: Supplementary file 1 — The results of multiple regression analyses excluding the cases with vitreous hemorrhage are shown in supplementary Table. [file 3427319.f1.docx]

| **Supplementary table**: Correlation between the expression levels of hsa-miR-148a-3p and clinical parameters in patients with retinal detachment (excluding the cases with vitreous hemorrhage, n = 22) | | |
| --- | --- | --- |
| Parameter | β | *P*-value |
| area of retinal break (pixels) | 0.746 | <0.001 |
| time from onset of RRD to vitrectomy (days) | 0.323 | 0.014 |
| range of retinal detachment (degrees) | 0.013 | 0.925 |

RRD, rhegmatogenous retinal detachment
